# Monitoring of blood glucose after pediatric kidney transplantation: a longitudinal cohort study

**DOI:** 10.1007/s00467-022-05669-0

**Published:** 2022-07-11

**Authors:** Doaa M. Salah, Mona Hafez, Ftaina I. Fadel, Yasmen Ahmed Said Selem, Noha Musa

**Affiliations:** 1 Pediatric Department, Faculty of Medicine, Cairo University, Cairo, Egypt; 2 Pediatric Nephrology & Transplantation Units, Cairo University Children Hospital, Cairo, Egypt; 3 Diabetes, Endocrine & Metabolism Pediatric Unit, Cairo University Children Hospital, Cairo, Egypt; 4 Ministry of Health, El Sahel Teaching Hospital, Cairo, Egypt

**Keywords:** Children, Glucose metabolism, Monitoring, Transplantation

## Abstract

**Background:**

Glucose metabolism after kidney transplantation (KT) is highly dynamic with the first post-transplantation year being the most critical period for new-onset diabetes after transplantation (NODAT) occurrence. The present study aimed to analyze dynamics of glucose metabolism and report incidence/risk factors of abnormal glycemic state during the first year after KT in children.

**Methods:**

Twenty-one consecutive freshly transplanted pediatric kidney transplant recipients (KTRs) were assessed for fasting plasma glucose (FPG) and oral glucose tolerance test (OGTT) weekly for 4 weeks, then every 3 months for 1 year.

**Results:**

Interpretation of OGTT test showed normal glucose tolerance (NGT) in 6 patients (28.6%) while 15 (71.4%) experienced impaired fasting glucose (IFG) and/or impaired glucose tolerance (IGT) at any time point of monitoring. Seven patients had NODAT, for which three needed insulin therapy. Hyperglycemia onset was 7.8 ± 13.12 weeks (median (range) = 1 (0–24) week) after KT. Percent of patients with abnormal OGTT was significantly more than that of IFG (38.1% vs. 71.4%, *p* = 0.029). Patients with abnormal glycemic state had significantly elevated trough tacrolimus levels at 6 months (*p* = 0.03). Glucose readings did not correlate with steroid doses nor rejection episodes while positively correlating with tacrolimus doses at 3 months (*p* = 0.02, CC = 0.73) and 6 months (*p* = 0.01, CC = 0.63), and negatively correlating with simultaneous GFR at 9 months (*p* = 0.04, CC =  − 0.57).

**Conclusions:**

Up to two thirds of pediatric KTRs (71.4%) experienced abnormal glycemic state at some point with peak incidence within the first week up to 6 months after KT. OGTT was a better tool for monitoring of glucose metabolism than FPG. Abnormal glycemic state was induced by tacrolimus and adversely affected graft function.

**Graphical abstract:**

A higher resolution version of the Graphical abstract is available as Supplementary information. 
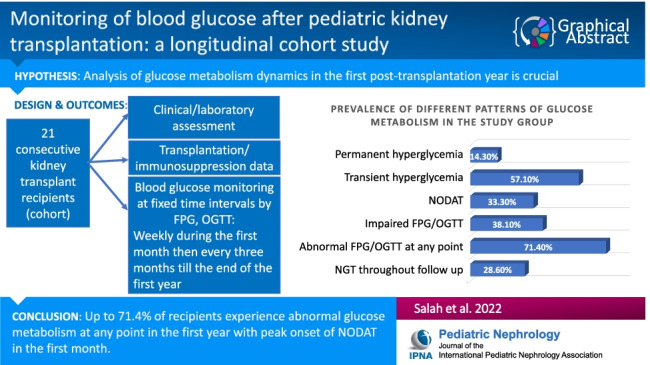

**Supplementary Information:**

The online version contains supplementary material available at 10.1007/s00467-022-05669-0.

## Introduction

Kidney transplantation (KT) is the ideal treatment for kidney failure, with better quality of life and lower risk of mortality compared with dialysis [1]*.* However, KT is not a complication-free modality of kidney replacement therapy (KRT). One of the important complications after KT is the development of new-onset diabetes mellitus after transplant (NODAT), which affects patient and kidney survival, and is associated with increased risk of cardiovascular (CV) morbidity [2].

There is wide variation in the reported incidence of NODAT in children (3–20%) due to differences in the use of diagnostic criteria, observation period, and immunosuppressive drugs with variable risk factors [3].

The first year after KT is the most critical as regards occurrence of NODAT, with reported double long-term mortality in adults who developed NODAT within their first KT year [4]. Moreover, glucose metabolism was reported to be highly dynamic, even several years after KT as kidney transplant recipients (KTRs) might flux into and out of the pre-diabetic state during their follow-up [5].

Disorders in glucose metabolism are silent and serious adverse events of KT in children, which necessitate screening, early detection, and appropriate treatment [6]*.* The dynamic nature of glucose metabolism after KT provides a continuous opportunity for intervention aiming to reduce diabetes-associated complications.

The aim of the present study was to investigate the incidence and potentially modifiable risk factors of abnormal glycemic state, and to analyze the dynamics of glucose metabolism during the first post-transplantation year in Egyptian children and adolescents.

## Patients and methods

The current study was a prospective longitudinal cohort study that included 21 consecutive pediatric KTRs. Recruited patients underwent KT in the Kidney Transplantation Unit and were followed up at the Kidney Transplantation Outpatient Clinic, Cairo University Children Hospital (CUCH) (Abu El Reech El Mounira). The study was conducted over a period of 2 years (2015–2017). An informed consent for enrollment in the study was obtained from patients’ legal guardians. The protocol of the study was approved by the Research Ethics Committee of the Pediatric Department, Faculty of Medicine, Cairo University (N: I-150315).

Included patients were all freshly transplanted recipients of a living related renal graft (according to national and hospital regulations), aged between 6 and 14 years, and were committed to follow-up for at least 1 year after KT.

### Baseline demographic, clinical, and transplantation related data

Basic data were collected at enrollment in the study, including age, sex, family history of diabetes or kidney disease, original kidney disease, comorbidities, pre-transplantation dialysis duration, and pre-transplantation treatment with steroids or lipid-lowering agents.

Clinical assessment was performed for all patients (*n* = 21) including weight and height measurements, as well as estimation of weight SDS and height SDS [7]. Body mass index (BMI) was calculated by using the formula BMI = weight (kg)/height (m^2^). Blood pressure (BP) percentiles were determined according to American Academy of Pediatrics (AAP) Clinical Practice Guidelines for Screening and Management of High Blood Pressure in Children and Adolescents [8] and were categorized as following: (1) normal BP, < 90th percentile; (2) elevated BP, ≥ 90th percentile to < 95th percentile or 120/80 mm Hg to < 95th percentile (whichever is lower); (3) stage 1 hypertension (HTN), ≥ 95th percentile to < 95th percentile + 12 mmHg, or 130/80 to 139/89 mm Hg (whichever is lower); (4) stage 2 HTN, ≥ 95th percentile + 12 mm Hg, or ≥ 140/90 mm Hg (whichever is lower).

Transplantation-related data were reviewed and patients were observed during follow-up assessments through their first post-transplantation year. Collected data were in the form of donor age/relationship, early graft function (in terms of decline of serum creatinine and achieving adequate urine output), and immunosuppressive medications including number/doses of pulse methylprednisolone received, cumulative steroid doses, trough levels of calcineurin inhibitors (CNIs; cyclosporine (CsA) or tacrolimus), and type of antiproliferative agent (mycophenolic acid (MPA), enteric coated mycophenolate (MPA/Na), or everolimus).

Steroids were administered as a part of induction therapy and then tapered gradually according to center guidelines as follows: intravenous methylprednisolone is given in 5–10 mg/kg (150–250 mg/m^2^) up to 250 mg/dose on the night before the operation, the time of induction of anesthesia, the time of de-clamping, and 6 h postoperatively, and the same dose is given once on a day following the operation. Steroids were then gradually tapered, converted to oral when tolerated in a dose of 2–3 mg/kg/day then reduced, targeting 15–20 mg/m^2^ by postoperative day 14. After the first post-transplantation month, dose was gradually tapered down to 2.5–7.5 mg/day at 6–12 months. Patients with acute rejection episodes (*n* = 8) received extra pulse methylprednisolone (250 mg/m^2^/dose for 3 successive days) followed by rapid tapering of oral steroids to or just above the maintenance regular dose.

Tacrolimus was administrated initially in a dose of 0.15 mg/kg/day in 2 divided doses. Tacrolimus dose was adjusted to therapeutic ranges based on drug-level monitoring, with target trough level of 10–12 ng/ml in the first month, 8–10 ng/ml until 3 months, 7–8 until 6 months, and 6–7 until the end of the first year post transplantation.

Acute graft dysfunction (AGD) was defined as rise of serum creatinine > 25% of the baseline level [9]. Presumed acute rejection (PRAR) was defined as AGD which is clinically diagnosed as acute rejection (after exclusion of other causes of AGD such as infection and dehydration) and medically treated by pulse methylprednisolone therapy, but kidney biopsy was not performed or did not show evidence of rejection [10]. Biopsy-proven acute rejection (BPAR) was defined as AGD accompanied by pathological evidence of rejection. First-line anti-rejection therapy was in the form of pulse methylprednisolone (150–250 mg/m^2^) for three successive days followed by rapid tapering of oral steroids for all acute rejection (AR) episodes. AR was considered to be steroid resistant if there was no response within 5–7 days after the first dose. Further treatment of AR was based on pathological findings of the kidney biopsy with acute T-cell-mediated rejections (*n* = 2) being treated with T-cell depleting therapy and antibody-mediated rejection (ABMR) (*n* = 3) being treated with plasma exchange, IVIG, and rituximab.

### Assessment of glucose profile

All patients were monitored for their glycemic state since their first postoperative week until the end of their first post-transplantation year. Blood samples were collected by peripheral venous puncture after 8 h of fasting, then fasting plasma glucose (FPG) was withdrawn followed by oral ingestion of 1.75 g/kg to a maximum dose of 75 g, and blood samples for glucose measurement were obtained every 30 min until 2 h post-glucose intake to assess oral glucose tolerance test (OGTT).

All patients were screened for pre-transplantation diabetes by review of pre-transplantation random blood sugar (RBS), performing fasting and 2-h postprandial blood sugar as well as assessing HBA1c. Patients with FPG above 126 mg/dl, postprandial glucose above 200 mg/dl, and/or HbA1c > 6.5% (denoting diabetes) were excluded from the study. FPG and OGTT were performed within the first week after transplantation then were assessed weekly for 4 weeks, then every 3 months for 1 year.

The American Diabetic Association (ADA) interpretation criteria of FPG and 2-h plasma glucose levels of the OGTT [11] were used for interpretation of measured values of plasma glucose as follows:

Normal glucose tolerance (NGT) was defined as FPG <100 mg/dl and 2-h glucose levels of OGTT <140 mg/dl. Impaired fasting glucose (IFG) was defined as FPG levels 100–125 mg/dl. Impaired glucose tolerance (IGT) was defined as 2-h glucose values 140–199 mg/dl. Diabetes mellitus (DM) was defined as FPG ≥126 mg/dl and/or second-hour glucose values during OGTT ≥200 mg/dl.

The definition of NODAT in the current study was based upon diabetes definition by ADA in absence of evidence of pre-transplantation DM. Permanent NODAT was defined as being on anti-diabetic medication and/or FPG ≥ 126 mg/dl at 1 year, while transient NODAT was defined as FPG < 126 mg/dl at 1-year assessment without anti-diabetic medications.

Separated simultaneous blood samples were tested for assessment of graft function (serum creatinine). Estimated glomerular filtration rate (eGFR) was calculated regularly (ml/min/1.73 m^2^) with follow-up assessments using Schwartz formula [12] as follows: GFR = 0.413 × (height/serum creatinine) with height expressed in centimeters and serum creatinine in milligrams per deciliter. Serum calcium, phosphorus alkaline phosphatase, and cholesterol levels were assessed at 6 months of follow-up of all patients.

**Fig. 1:**
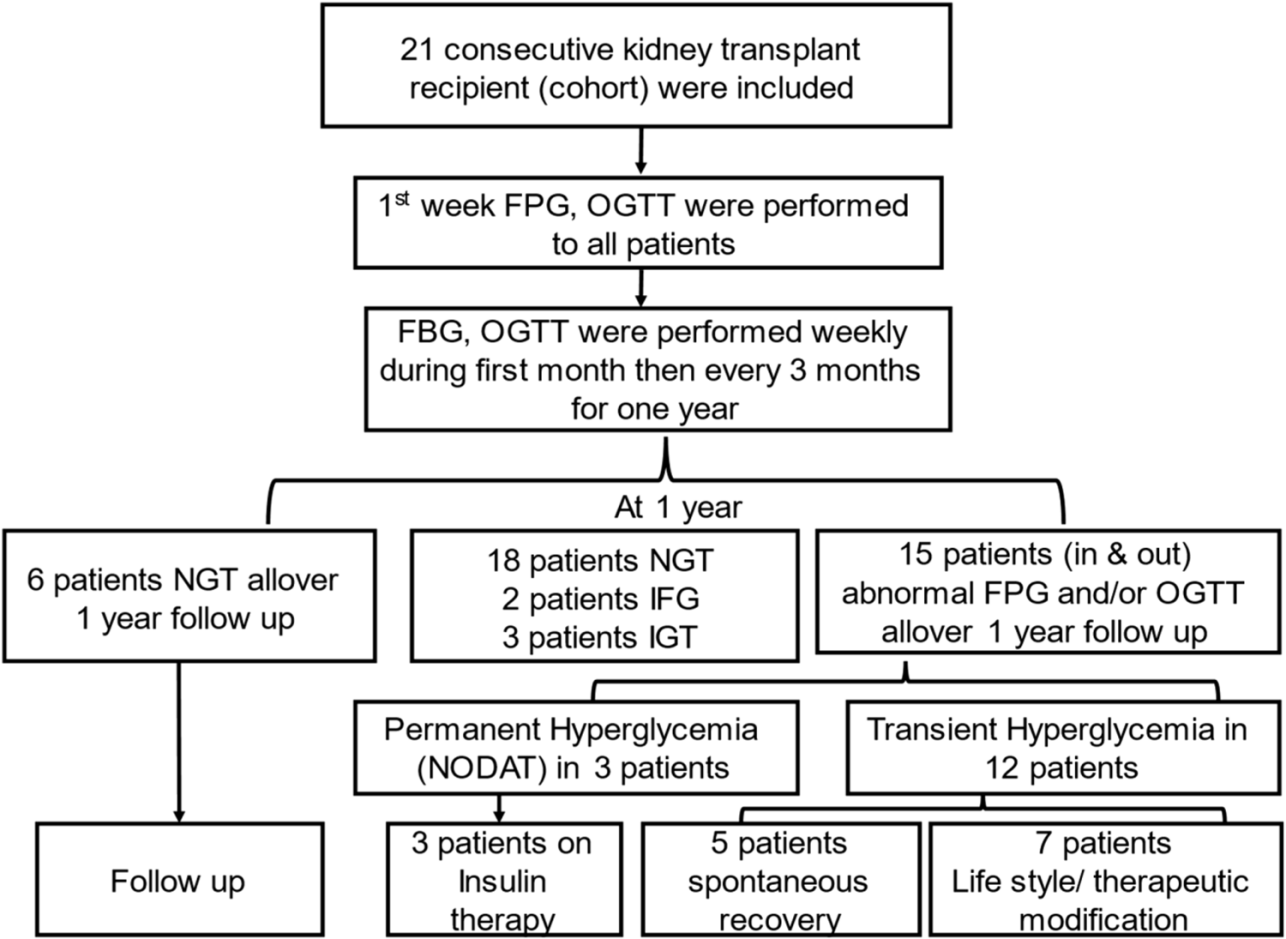
Flow chart of monitoring of glucose metabolism, interpretation of glucose readings, and management in the study group (*n* = 21)

Patients’ enrollment in the study, follow-up assessment, interpretation of OGTT, and further management are summarized in Fig. 1.

### Statistical analysis

All statistical calculations were done using computer program IBM SPSS (Statistical Package for the Social Science; IBM Corp, Armonk, NY, USA) release 22 for Microsoft Windows. Nominal data were expressed as frequency and percentage and were compared using chi-square (*χ*^2^) test. Numerical data were expressed as mean ± standard deviation (SD) and were compared using *t* test and Wilcoxon signed-rank test. Non-parametric data were expressed as median and interquartile range (IQR) and were compared using Mann–Whitney (*U* test) for comparing two groups and Kruskal–Wallis test in comparing more than two groups. For comparing categorical data, *χ*^2^ test was performed. Exact test was used instead when the expected frequency was less than 5. Correlation between various variables was done using Spearman rank correlation equation. *P* values less than 0.05 were considered statistically significant.

## Results

The mean age of the study group (*n* = 21) was 10.7 ± 9.89 years with male-to-female ratio of 2:1 (14 males and 7 females). Thirteen patients (61.9%) had a family history of type 2 DM and 5 patients (23.8%) had a family history of similar kidney disease. Primary kidney disease of the study group was obstructive uropathy in 6 (28.6%) patients, unknown kidney failure in 5 (23.8%) patients, juvenile nephronophthisis in 3 (14.3%) patients, glomerulopathies in 5 (23.8%) patients, Caroli disease in 1 (5%) patient, and atypical hemolytic uremic syndrome (HUS) in 1 (5%) patient. No comorbidities were reported among the study group apart from hepatosplenomegaly with normal liver functions in the patient with Caroli disease. Patients with glomerulopathies (*n* = 5) received steroid therapy for a duration ranging between 3 and 6 months that was withdrawn at least 6 months before need for initiation of regular dialysis. No patients in the study group received a lipid-lowering agent.

## Transplantation-related data

All patients were recipients of living related donor renal graft (donors were either mother (62%), father (23%), brother (4.7%), uncle (4.7%), or aunt (4.7%) of the recipients). Early graft function was excellent (i.e., immediate urine flow > 1 ml/kg/h and gradual decline of serum creatinine to < 1 mg/dl) in 17 (81%) patients while 4 (19%) patients experienced transient oliguria due to acute tubular necrosis (ATN). Thirteen patients (62%) suffered from postoperative HTN (BP was controlled (stage 1 HTN) in 11 patients and uncontrolled (stage 2 HTN) on medications in 2 patients). Demographic, clinical, and transplantation-related data of the studied cohort are summarized in Table 1.

**Table 1:** Demographic, clinical, and transplantation-related data of patients

	Total cohort (*n* = 21)	Patients with normal OGTT (*n* = 6)	Patients with abnormal OGTT (*n* = 15)*	*P* value (normal versus abnormal OGTT)
Age (years) at TX	10.7 ± 9	11.83 (± 3.37)	10.37 (± 2.53)	0.29
Weight SDS at TX	–1.15 ± 1.8712	1.52 (± 1.14)	–1.0 (± 2.11)	0.85
Height SDS at TX	–7.71 ± 2.54	–2.14 (± 1.25)	–1.85 (± 2.93)	0.85
BMI SDS at TX	–0.52 ± 1.87	–0.52 (± 1.87)	–0.51 (± 1.94)	0.91
Dialysis duration (years)	1.99 ± 2.92	1.583** ± **1.272	2.06** ± **3.39	0.61
Donor age (years)	36.67 ± 5.102	36.50 ± 3.21	36.73 ± 5.79	0.39
Family H/O of DM type 2	13 (61.9%)	4 (66.7%)	9 (60%)	1
Pre-TX RBS (mg/dl)	100.57 ± 21.91	99.12 ± 27.6	100.75 ± 11.79	0.216
Pre-TX cholesterol	213.29 ± 81.45	187.25 ± 29.76	250.37 ± 86.38	0.091
# BP				
Normal BP	6 (28.6%)	4 (57.1%)	2 (14.3%)	0.58
Elevated BP	0 (0%)	0 (0%)	0 (0%)	
Stage 1 hypertension	13 (61.9%)	3 (82.9%)	10 (71.4%)	
Stage 2 hypertension	2 (9.5%)	0 (0%)	2 (14.3%)	
S. Calcium at 6 months	9.63 ± 2.87	9.7 ± 0.5	9.45 ± 0.74	0.232
S. Phosphorus at 6 months	3.76 ± 0.72	4.13 ± 0.89	4.13 ± 0.51	0.341
S. ALP at 6 months	596.5 ± 876.82	300.3 ± 234.2	235.3 ± 870.8	0.185
S. Cholesterol 6 months	182.4 ± 41.73	134.5 ± 85	215.6 ± 49.8	0.08
S. Creatinine (mg/dl) 6 months	0.733 ± 0.19	0.93 ± 0.25	1.03 ± 0.49	0.432
e GFR (ml/min/1.73 m^2^) 6 months	82.16 ± 20.24	62.8 ± 15.3	66.05 ± 32.6	0.474
S. Creatinine (mg/dl) at 1 year	0.95 ± 0.37	1 ± 0.459	1.07 ± 0.55	0.08
eGFR (ml/min/1.73 m^2^) at 1 year	70.7 ± 23.5	75.23 ± 25.43	59.84 ± 13.5	0.17

*TX* transplantation, *RBS* random blood sugar, *BMI* body mass index, *SDS* standard deviation score, *H/O* history, *DM* diabetes mellitus, *BP* blood pressure, *S* serum, *eGFR* estimated glomerular filtration rate, *ALP* alkaline phosphatase

^*^Abnormal OGTT includes patients with IFG, IGT, and DM and those who developed any of them at any assessment point interval

^#^Blood pressure data point to interpretation of the 6-month BP assessment data

As illustrated in Table 1, patients with NGT (*n* = 6) all through the follow-up period of blood glucose assessment (1 year post transplantation) were compared to patients with abnormal OGTT (including patients with changing pattern between NGT, IFG, IGT, or DM) (*n* = 15), in order to identify the risk factors for developing abnormal OGTT after KT. No significant difference was detected between patients with normal and abnormal OGTT as regards primary kidney disease, donor relationship or early graft function (*p* = 0.43, 0.77, and 1, respectively). Although patients with family history of type 2 DM were mainly in the abnormal OGTT group, the difference in this regard between patients with and without normal OGTT did not reach statistical significance (13 patients with 4 of them having normal OGTT and 9 having abnormal OGTT, *p* = 1).

### Immunosuppression and rejection data

All patients received antibody induction (Ab) therapy except for one patient with low immunological risk in whom Ab induction was not indicated. All patients received classic triple therapy maintenance immunosuppression protocol consisting of steroids, CNI, and antiproliferative agent.

CNI was in the form of CsA in 5 patients and tacrolimus in 16 patients during the first 6 months after KT. Three patients had their CNI changed from CsA to tacrolimus during the next 6 months due to CsA-associated adverse effects. Antiproliferative agent was in the form of MPA in 6 patients and MPA/Na in 15 patients. Everolimus was introduced with low CNI doses 6 months after KT in three patients and continued until the end of the first year in one patient, while classic triple therapy was resumed in the other two patients. No significant differences were seen between patients with normal OGTT (*n* = 6) and abnormal OGTT (*n* = 15) as regards simultaneous and cumulative doses of steroids at 3, 6, and 12 months (*p* > 0.05). Patients with abnormal OGTT (*n* = 15) had significantly elevated mean trough levels of tacrolimus during the second half of their first post-transplantation year (*p* = 0.03) (Table 2).

**Table 2:** Immunosuppression and rejection data of patients with normal and impaired OGTT

			Patients with normal OGTT (*n* = 6)	Patients with abnormal OGTT (*n* = 15)*	*P* value
ATG induction immunotherapy	*n* = 8	*n* (%)	3 (50%)	5 (33.3%)	0.864
Basiliximab induction immunotherapy	*n* = 12	*n* (%)	3 (50%)	9 (60%)	0.528
Steroid (mg/m^2^/day) at 3 mo	*n* = 21	Mean ± SD	25.55 ± 11.134	22.43 ± 8.44	0.69
Steroid (mg/m^2^/day) at 6 mo	*n* = 21	Mean ± SD	22.81 ± 9.5895	32.04 ± 36.36	0.82
Steroid (mg/m^2^/day) at 12 mo	*n* = 21	Mean ± SD	19.50 ± 8.91	24.01 ± 28.62	0.54
Cumulative steroids (mg/m^2^/day) at 3 mo	*n* = 21	Mean ± SD	635.817 ± 406.9	814.97 ± 849.73	0.94
Cumulative steroids (mg/m^2^/day) at 6 mo	*n* = 21	Mean ± SD	726.25 ± 548.17	847.12 ± 892.33	0.76
Cumulative steroids (mg/m^2^/day) at 12 mo	*n* = 21	Mean ± SD	779.43 ± 710.67	1033.53 ± 1116.91	0.64
Cyclosporine (mg/day) at 3 mo	*n* = 8	Mean ± SD	260 ± 34.88	231.57 ± 57.22	0.34
Cyclosporine (mg/day) at 6 mo	*n* = 8	Mean ± SD	250 ± 20.46	226.7 ± 48.15	0.38
Cyclosporine (mg/day) at 12 mo	*n* = 5	Mean ± SD	225 ± 0	229.65 ± 30.15	0.48
Cyclosporine level (0–6) (ng/ml)	*n* = 8	Mean ± SD	227.36 ± 95.09	329.44 ± 115.15	0.2
Cyclosporine level (6–12) (ng/ml)	*n* = 5	Mean ± SD	58.45 ± 74.32	120.78 ± 90.34	0.44
Tacrolimus (mg/day) at 3 mo	*n* = 13	Mean ± SD	5.69 ± 1.01	6.79 ± 1.55	0.14
Tacrolimus (mg/day) at 6 mo	*n* = 13	Mean ± SD	5.45 ± 0.86	6.89 ± 1.79	0.14
Tacrolimus (mg/day) at 12 mo	*n* = 16	Mean ± SD	4.63 ± 0.86	5.92 ± 2.82	0.61
Tacrolimus level (0–6 mo))ng/ml)	*n* = 13	Mean ± SD	5.78 ± 3.72	8.59 ± 2.56	0.21
Tacrolimus level (6–12 mo) (ng/ml)	*n* = 16	Mean ± SD	4.0 ± 2.78	8.34 ± 0.94	**0.03**
MPA/Na (mg/day) at 3 mo	*n* = 15	Mean ± SD	716.20 ± 151.56	891.32 ± 362.56	0.56
MPA/Na (mg/day) at 6 mo	*n* = 15	Mean ± SD	782.76 ± 195.43	782.76 ± 406.25	0.55
MPA/Na (mg/day) at 12 mo	*n* = 16	Mean ± SD	733.60 ± 125.71	665.96 ± 112.00	0.46
Everolimus (mg/kg/day) at 6 mo	*n* = 3	Mean ± SD	1.50 ± 0	1.49 ± 0.09	1.00
Presumed acute rejection (PRAR)	*n* = 8	*n* (%)	3 (50%)	5 (33.3%)	0.466
Biopsy-proven acute rejection (BPAR)	*n* = 5	*n* (%)	1 (16.7%)	4 (26.7%)	0.157

*ATG* antithymocyte globulin, *MPA/Na* enteric coated mycophenolic acid

^*^Abnormal OGTT includes patients with IFG, IGT and DM and those who developed any of them at any assessment point interval

Eight patients (38%) experienced AGD during their follow-up visits and were diagnosed as PRAR, for which pulse methylprednisolone therapy was received and graft biopsy was performed. Pathological confirmation of rejection (BPAR) was achieved in five patients (23.8%). No significant difference was reported between patients with normal and abnormal OGTT as regards rejection data (Table 2).

### Monitoring of plasma glucose levels

OGTT results were interpreted over the first year post transplantation. NGT was reported in 6 patients (28.6%) within the study cohort. In 15 patients (71.4%); abnormal FPG and/or OGTT at any point of follow-up intervals was detected; 7 patients (33.3%) had plasma glucose readings compatible with NODAT and 8 patients (38.1%) with IFG and/ or IGT. Of patients with NODAT (*n* = 7), three patients (14.3%) needed insulin therapy and were categorized as having permanent hyperglycemia while four patients (19.1%) had transient hyperglycemia (Fig. 1).

Glycemic data delivered by FPG and OGTT are presented in Table 3. The percentage of patients with abnormal OGTT was more than those with IFG at all point intervals of assessment but without statistical significance (*p* > 0.05). Nevertheless, incidence of abnormal glycemic state documented by interpretation of OGTT was significantly different from that reported by FPG among the study cohort (normal/IFG = 13/8 versus normal/abnormal OGTT = 6/15, *p* = 0.0299).

**Table 3:** Glycemic data delivered by FPG and OGTT in the study cohort (*n* = 21)

Timing after TX	Glucose readings by OGTT (mean ± SD)		OGTT max (mean ± SD)	Abnormal FPG *N* (%)		Abnormal OGTT *N* (%)		*P* value
1 week	G0	92.84 ± 25.48	151.11 ± 34.89	Total IFG DM	3 (14.3%) 2 (9.5%) 1(4.7%)	Total IGT DM	4 (19.1%) 2 (9.5%) 2 (9.5%)	0.679
	G30	126.48 ± 27.1						
	G60	134.89 ± 46.78						
	G90	128.2 ± 28.1						
	G120	113.05 ± 27.38						
2 weeks	G0	90.05 ± 22.35	185.52 ± 70.22	Total IFG DM	6 (28.6%) 3 (14.3%) 3 (14.3%)	Total IGT DM	7 (33.3%) 1 (4.7%) 6 (28.6%)	0.739
	G30	163.5 ± 70.51						
	G60	171.4 ± 68.73						
	G90	162.2 ± 57.43						
	G120	145.85 ± 52.8						
3 weeks	G0	91.78 ± 14.88	143.33 ± 29.05	Total IFG DM	3 (14.3%) 1 (4.7%) 2 (9.5%)	Total IGT DM	5 (23.8%) 3 (14.3%) 2 (9.5%)	0.432
	G30	122.33 ± 19.52						
	G60	124.5 ± 16.7						
	G90	123.56 ± 22.59						
	G120	116.78 ± 36.56						
1 month	G0	96.38 ± 18.99	139 ± 21.98	Total IFG DM	3 (14.3%) 3 (14.3%) 0 (0%)	Total IGT DM	6 (28.6%) 5 (23.8%) 1 (4.7%)	0.259
	G30	129.86 ± 14.23						
	G60	130.38 ± 18.03						
	G90	120.86 ± 24.22						
	G120	111.48 ± 34.74						
3 months	G0	90.15 ± 10.28	146.83 ± 28.15	Total IFG DM	4 (19.1%) 4 (19.1%) 0 (0%)	Total IGT DM	6 (28.6%) 6 (28.6%) 0 (0%)	0.469
	G30	143.4 ± 28.1						
	G60	137.55 ± 29.8						
	G90	122.6 ± 24.69						
	G120	113.4 ± 26.46						
6 months	G0	88.89 ± 11.44	137.53 ± 25.91	Total IFG DM	3 (14.3%) 3 (14.3%) 0 (0%)	Total IGT DM	6 (28.6%) 6 (28.6%) 0 (%)	0.259
	G30	126.53 ± 27.22						
	G60	126.26 ± 20.48						
	G90	117.78 ± 25.76						
	G120	111.42 ± 30.85						
9 months	G0	90.21 ± 14.72	126.67 ± 25.78	Total IFG DM	3 (14.3%) 3 (14.3%) 0 (0%)	Total IGT DM	4 (19.1%) 4 (19.1%) 0 (%)	0.259
	G30	117.29 ± 24.8						
	G60	115.64 ± 20.85						
	G90	110.5 ± 20.49						
	G120	104.21 ± 18.79						
12 months	G0	88.25 ± 19.04	130.8 ± 19.46	Total IFG DM	2 (9.5%) 2 (9.5%) 0 (0%)	Total IGT DM	3 (14.3%) 3 (14.3%) 0 (0%)	0.634
	G30	112.58 ± 22.91						
	G60	127.92 ± 24.36						
	G90	124.5 ± 17.02						
	G120	114.58 ± 16.42						

*TX* transplantation, *IFG* impaired fasting glucose, *IGT* impaired glucose tolerance, *DM* diabetes mellitus

**Fig. 2:**
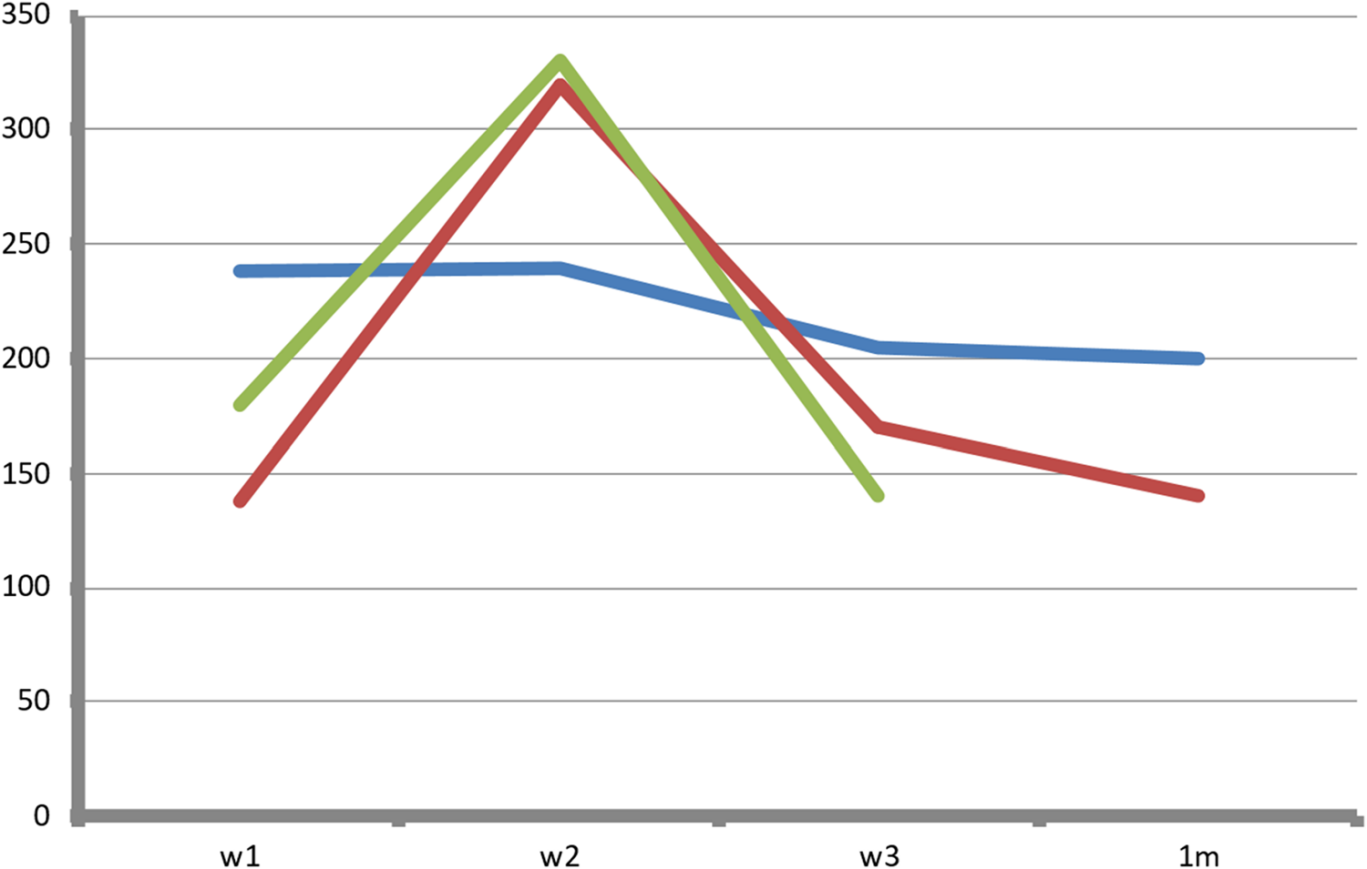
OGTT max (mg/dl) in permanent hyperglycemia patients (*n* = 3) before starting insulin therapy

Onset of occurrence of hyperglycemia among abnormal OGTT patients (*n* = 15) was 7.8 ± 13.12 weeks after KT (median 1 week with range between 0 and 24 weeks). Patients with NODAT (*n* = 7) had their onset of DM within the first week (in 2 patients) and second week (in 5 patients) after KT. Figure 2 illustrates maximum glucose reading (mg/dl) by OGTT in patients with permanent hyperglycemia (*n* = 3) before starting insulin therapy. Patients with permanent hyperglycemia received multiple daily insulin injections (basal-bolus regimen) in the form of insulin glargine and insulin aspart in a dose ranging between 0.3 and 0.8 IU/kg/day (mean ± SD = 0.60 ± 0.27 U/kg/day). HbA1c was assessed every 3 months in those patients and it ranged between 4.2% and 9.6% (mean of 5.83** ± **1.42%).

**Fig. 3:**
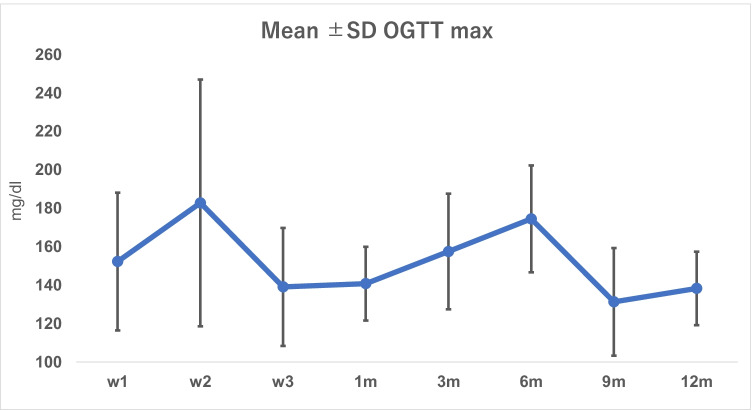
Mean ± SD of OGTT max (mg/dl) in transient hyperglycemia patients (*n* = 12)

Patients with transient hyperglycemia (*n* = 12) had their onset of impaired OGTT starting between the first week and 6 months after KT (Fig. 3). On follow-up of patients with transient hyperglycemia, seven patients (33.3%) reverted to normal by lifestyle and therapy modifications and five patients (23.8%) reverted spontaneously within the first year after transplantation (Fig. 1). Glycemic status of included patients throughout the follow up period is detailed in Table 4.

**Table 4:** Individual glycemic status at different points throughout the follow-up

	**1 week**	**2 weeks**	**3 weeks**	**1 month**	**3 months**	**6 months**	**9 months**	**1 year**
Patient 1	NGT	**DM**	NGT	NGT	NGT	NGT	NGT	NGT
Patient 2	**IFG,IGT**	NGT	NGT	NGT	NGT	**IFG,IGT**	**IFG,IGT**	**IFG,IGT**
Patient 3	NGT	NGT	NGT	**IFG**	**IFG**	NGT	NGT	NGT
Patient 4	NGT	NGT	NGT	NGT	**IFG,IGT**	**IFG,IGT**	**IFG,IGT**	**IFG**
Patient 5	NGT	NGT	NGT	NGT	NGT	NGT	NGT	NGT
Patient 6	NGT	NGT	NGT	NGT	NGT	NGT	NGT	NGT
Patient 7	NGT	NGT	NGT	NGT	**IGT**	NGT	NGT	NGT
Patient 8*	**DM**	**DM**	**IFG**	NGT	NGT	**IFG**	**IFG**	NGT
Patient 9	NGT	NGT	NGT	NGT	NGT	NGT	**IGT**	**IGT**
Patient 10	NGT	**IGT**	NGT	NGT	**IGT**	**IGT**	NGT	NGT
Patient 11	NGT	NGT	NGT	NGT	NGT	NGT	NGT	NGT
Patient 12	NGT	NGT	NGT	NGT	NGT	NGT	NGT	NGT
Patient 13	NGT	NGT	NGT	NGT	**IFG,IGT**	NGT	NGT	NGT
Patient 14*	NGT	**DM**	**DM**	**DM**	**IFG,IGT**	**IGT**	NGT	NGT
Patient 15	NGT	NGT	NGT	NGT	NGT	NGT	NGT	NGT
Patient 16	NGT	**DM**	**DM**	**IGT**	NGT	NGT	NGT	NGT
Patient 17	**IFG,IGT**	NGT	NGT	**IFG**	NGT	**IGT**	NGT	NGT
Patient 18*	**DM**	**DM**	**IFG,IGT**	**IFG**	NGT	NGT	NGT	NGT
Patient 19	NGT	NGT	NGT	NGT	NGT	NGT	NGT	NGT
Patient 20	NGT	**DM**	**IGT**	**IGT**	NGT	NGT	NGT	NGT
Patient 21	NGT	**DM**	NGT	NGT	NGT	NGT	NGT	NGT

*NGT* normal glucose tolerance, *IFG* impaired fasting glucose, *IGT* impaired glucose tolerance. Bold entries refer to any form of abnormal glucose metabolism reported for each patient

Controlled on insulin * NODAT on insulin therapy

### Correlations of OGTT readings

No significant correlation was found between OGTT readings and demographic, clinical, or disease/transplantation-related data. No significant correlation was detected between steroid doses and OGTT readings. A significant positive correlation between OGTT readings and tacrolimus doses at 3 and 6 months was reported (*p* = 0.03 and 0.01, respectively). OGTT readings negatively correlated with eGFR at 9-month follow-up (*p* = 0.04, *r* =  − 0.57) (Table 5).

**Table 5:** Correlation between abnormal OGTT and different variables

	OGTT		
	Number	*P* value	Correlation coefficient (CC)
Age (years)	21	0.3	–0.23
BMI SDS	21	0.91	0.03
Donor age (years)	21	0.39	0.19
Dialysis duration (years)	21	0.62	–0.11
Cholesterol (mg/dl)	21	0.93	–0.02
Mean steroid 3 mo	21	0.84	0.05
Mean cyclosporine 3 mo	8	1.0	0.0
Mean tacrolimus 3 mo	**13**	**0.02**	**0.63**
Mean MPA/Na 3 mo	**15**	**0.03**	**0.91**
Mean steroid 6 mo	21	0.56	–0.14
Mean cyclosporine 6 mo	8	0.5	0.28
Mean tacrolimus 6 mo	**13**	**0.01**	**0.73**
Mean MPA/Na 6 mo	15	0.72	–0.1
Mean everolimus 6 mo	3	0.33	0.87
Mean steroid 12 mo	21	0.87	0.05
Mean cyclosporine 12 mo	5	0.89	0.11
Mean tacrolimus 12 mo	16	0.64	–0.18
Mean MPA/Na 12 mo	16	0.93	0.04
Cumulative steroid at 1 mo	21	0.09	–0.38
Cumulative steroid at 3 mo	21	0.59	–0.13
Cumulative steroid at 6 mo	21	0.36	–0.22
Cumulative steroid at 9 mo	21	0.18	–0.39
Cumulative steroid at 12 mo	21	0.20	0.39
GFR at 1 week	21	0.44	0.19
GFR at 2 weeks	21	0.05	0.56
GFR at 3 weeks	21	0.41	–0.2
GFR at 1 mo	21	0.07	0.4
GFR at 3 mo	21	0.79	0.06
GFR at 6 mo	21	0.17	0.33
GFR at 9 mo	**21**	**0.04**	**–0.57**
GFR at 12 mo	21	0.87	0.05

*BMI SDS* body mass index standard deviation score, *MPA/Na* enteric coated mycophenolic acid, *GFR* glomerular filtration rate

## Discussion

Hyperglycemia after transplantation has been reported to be associated with a variety of adverse outcomes [13]. Distinct categories of patients with hyperglycemia following organ transplant have been identified, including known pre-existing DM, persistent hyperglycemia, and NODAT [14]. In the present study, we aimed to monitor blood glucose by FPG and OGTT at fixed time intervals through the first year after KT in a pediatric cohort followed up at CUCH.

In the current study, we reported an overall frequency of abnormal glucose metabolism up to 71.4% with 33.3% of patients having NODAT. Most of the studies conducted for similar purpose were cross-sectional in nature rather than longitudinal ones, which explains our high incidence when compared to previously published pediatric data (3–26.2%) [15–18]. Longitudinal adult reports showed similar results to the present study, with only about one third of their patients showing normal glucose regulation [5]. At fixed point intervals, however, we reported similar frequency of glucose abnormalities after transplantation to previous reports. For example, abnormal FPG and/or OGTT was present in 28.6% of patients at 1, 3, and 6 months after KT, with only 4.7% of patients having NODAT at 1 month. This frequency is in accordance with published data of a recent study conducted in our center with an overall frequency of abnormal glucose metabolism of 23.3% with 10% of patients having post-transplant diabetes mellitus (PTDM) [6]. Data provided by the current study proves that frequency of IFG and/or IGT has declined by the second half of the first post-transplantation year (up to 19.1% and 14.3% at 9 and 12 months, respectively, with no reported cases of NODAT). So, continuously high fluxes between states of glucose metabolism, with the state of individual patients deteriorating or improving over time, resulted in a high number of our incident patients.

In the present study, although the majority of patients with family history of DM developed abnormalities in glucose metabolism during follow-up (13 patients with family history of type 2 DM; 4 and 9 patients with and without NGT, respectively), it was not determined as a risk factor for development of hyperglycemia after KT (*p* = 1). Similarly, Porrini et al. did not find any relationship between family history of DM and NODAT [19]. In contrast to our work, Pham et al. found strong evidence suggesting that positive family history of DM among first-degree relatives was an important risk factor for development of PTDM in transplant recipients [20]. At the end of the day, NODAT is categorized as type 2 DM. However, the small sample size of this study limits the result validity in this regard.

In the present study, the onset of hyperglycemia was 7.8 ± 13.12 weeks (median (range) = 1 (0–24) week) after KT. An adult longitudinal follow-up study has been conducted at fixed point to assess glucose metabolism after KT. Guthoff and co-workers reported a persistent patient flux into and out of the pre-diabetic state reflecting the highly dynamic nature of glucose [5]. This fact is highly supported by our results (Table 4). However, we studied glucose state at earlier time intervals starting immediately after KT. Moreover, we found that all patients with NODAT (*n* = 7) have been diagnosed within the first post-transplantation month. This is well explained by the hyperglycemic effect of high doses of corticosteroids and CNI needed within the early postoperative period. Moreover, Hjelmesaeth et al. proposed a division of NODAT into three clinical forms with the first one occurring very early after transplantation due to insulin resistance secondary to high doses of corticosteroids [21].

The sensitivity of FPG in screening for NODAT has previously been questioned, while OGTT has been mooted as an alternative, but is inconvenient for all patients. In the present study, we report that the incidence of abnormal glycemic state diagnosed by OGTT was significantly higher than that diagnosed by FPG (38.1% vs. 71.4%, *p* = 0.0299). Our finding is well supported by data recently published from our center also. The authors concluded that using FPG levels as a screening tool will underdiagnose abnormalities of glucose metabolism after KT and that OGTT is the gold-standard method for assessment, not only after transplantation but also prior to it [6]. Similarly, previous studies conducted on adult transplant recipients reported the same finding [22, 23].

Among immunosuppressive drugs used in solid organ transplantation protocols, tacrolimus is characterized by the strongest diabetogenic effects [24]. In the present study, patients with abnormal glycemic state had significantly elevated trough tacrolimus levels at 6 months (*p* = 0.03). Our findings came in accordance with a report of a multi-center DIRECT trial where the authors described a significantly higher incidence of abnormal FPG 6 months after KT as well as of NODAT in patients treated using tacrolimus (34% vs. 26%, *p* = 0.046) [25]. Arafa and co-workers in their pediatric reports failed to find a significant difference between groups of normal and abnormal glucose metabolism regarding the type of CNI used; nevertheless, 85.7% of their patients with abnormal glucose metabolism were receiving tacrolimus and only 14.3% of them were receiving CsA with significantly higher tacrolimus trough values among those with abnormal glucose metabolism [6].

In the present study, glucose readings did not correlate with steroid doses nor rejection episodes, while they positively correlated with tacrolimus doses at 3 months (*p* = 0.02, CC = 0.73) and 6 months (*p* = 0.01, CC = 0.63). Moreover, we found that glucose readings negatively correlated with simultaneous eGFR at 9 months (*p* = 0.04, CC =  − 0.57) reflecting the probable negative impact of abnormalities in glucose metabolism after KT on graft survival. Abnormal glucose metabolism has been reported to impact allograft survival [26]. However, the presence of confounders that could also affect graft function limits interpretation of our results in this regard.

Our results show no significant association between steroid doses and NODAT which was unexpected since steroids are a well-established cause of hyperglycemia through several mechanisms. The effect of steroids on inducing hyperglycemia had been historically believed to be dose dependent [27]. However, in a study conducted by Burroughs et al., based on data from the USRDS registry, the authors reported that increasing steroid dosages potentiates the diabetogenic effect of tacrolimus while steroid dosage did not seem to affect the risk for NODAT in patients who receive CsA [28]. Owing to the fact that 24% of patients in the current study received cyclosporine, in addition to the small sample size and lack of control group on steroid-free protocol, our results in this regard may have some reservations.

The main limitation of this study is the small sample size. Also, lack of detailed glycemic data before transplantation and longer follow-up after KT could limit the data of this study. Further studies are planned to overcome these limitations.

In conclusion, abnormalities of glucose metabolism are highly prevalent after pediatric kidney transplantation to the extent that up to two thirds (71.4%) of pediatric KTRs experience abnormal glycemic state at any point within their first post-transplantation year. The peak incidence of NODAT was within the first month after KT, with IGT occurring with a peak from first week and up to 6 months after KT. Patient fluxes into and out of the pre-diabetic state reflect the highly dynamic nature of glucose metabolism during the observation period, with OGTT being a better tool for monitoring glucose metabolism than FPG. Abnormal glycemic state was induced by tacrolimus and could adversely affect graft function. Frequent monitoring of blood glucose by OGTT, especially during the early post-transplantation period (weekly in the first month, twice monthly in the second month, and monthly in the following 4 months), and lifestyle/immunosuppression modifications in patients with abnormal OGTT are recommended.

## Supplementary Information

Below is the link to the electronic supplementary material.

Graphical Abstract (PPTX 111 kb)

Supplementary file2 (DOCX 17 KB)

## Declarations

### Data Availability

The datasets used and/or analyzed during the current study are available from the corresponding author on reasonable request.

## References

[1] Sawinski D, Poggio ED (2021). Introduction to kidney transplantation: long-term management challenges. Clin J Am Soc Nephrol.

[2] Almardini R, Salaita G, Albderat J, Alrabadi K, Alhadidi A, Alfarah M (2019). Diabetes mellitus after pediatric kidney transplant. Exp Clin Transplant.

[3] Garro R, Warshaw B, Felner E (2015). New-onset diabetes after kidney transplant in children. Pediatr Nephrol.

[4] Cole EH, Johnston O, Rose CL, Gill JS (2008). Impact of acute rejection and new-onset diabetes on long-term transplant graft and patient survival. Clin J Am Soc Nephrol.

[5] Guthoffa M, Wagnera R, Weichbrodta K, Nadalind S, Königsrainerd A, Häringa H, Fritschea A, Heyne N (2017). Dynamics of glucose metabolism after kidney transplantation. Kidney Blood Press Res.

[6] Arafa N, Bazaraa HM, Sharaf El Din H, Hussein M, Salah DM (2021). Glucose tolerance in a cohort of Egyptian children after kidney transplantation. Diabetes Res Clin Pract.

[7] Ghali I, Salah N, Hussein F, Erfan M, El-Ruby M, Mazen I (2008) Egyptian growth curves for infants, children and adolescents. In: Satorio A, Buckler JMH, Marazzi N, editors. Greece nel mondo. Milan, Italy: Ferring Publisher

[8] Flynn JT, Kaelber DC, Baker-Smith CM, Blowey D, Carroll AE, Daniels SR, de Ferranti SD, Dionne JM, Falkner B, Flinn SK, Gidding SS, Goodwin C, Leu MG, Powers ME, Rea C, Samuels J, Simasek M, Thaker VV, Urbina EM, Subcommittee on screening and management of high blood pressure in children (2017). Clinical Practice Guideline for Screening and Management of High Blood Pressure in Children and Adolescents. Pediatrics.

[9] Hagras AM, Salah DM, Ahmed DH, Abd Elaala OK, Elghobary HAF, Fadel FI (2018). Serum soluble interleukin 2 receptor level as a marker of acute rejection in pediatric kidney transplant recipients. Nephron.

[10] Fleiner FL, Glander P, Neumayer HH, Budde K (2006). Reporting of rejection after renal transplantation in large immunosuppressive trials: biopsy-proven, clinical, presumed, or treated rejection?. Transplantation.

[11] American Diabetes Association (ADA) (2020). Improving care and promoting health in populations: standards of medical care in diabetes 2020. Diabetes Care.

[12] Schwartz GJ, Work DF (2009). Measurement and estimation of GFR in children and adolescents. J Am Soc Nephrol.

[13] Hecking M, Werzowa J, Haidinger M, Hörl WH, Pascual J, Budde K, Luan FL, Ojo A, de Vries AP, Porrini E, Pacini G, Port FK, Sharif A, Säemann MD, European-New-Onset Diabetes After Transplantation Working Group (2018). Novel views on new-onset diabetes after transplantation: development, prevention and treatment. Nephrol Dial Transplant.

[14] Gupta S, Pollack T, Fulkerson C, Schmidt K, Oakes DJ, Molitch ME, Wallia A (2018). Hyperglycemia in the posttransplant period: NODAT vs posttransplant diabetes mellitus. J Endocr Soc.

[15] Koshy SM, Guttmann A, Hebert D, Parkes RK, Logan AG (2009). Incidence and risk factors for cardiovascular events and death in pediatric renal transplant patients: a single center long-term outcome study. Pediatr Transplant.

[16] Buyan N, Bilge I, Turkmen MA, Bayrakci U, Emre S, Fidan K (2010). Post-transplant glucose status in 61 pediatric renal transplant recipients: preliminary results of five Turkish pediatric nephrology centers. Pediatr Transplant.

[17] Prokai A, Fekete A, Kis E, Reusz GS, Sallay P, Korner A (2008). Post-transplant diabetes mellitus in children following renal transplantation. Pediatr Transplant.

[18] Al-Uzri A, Stablein DM, Cohn AR (2001). Posttransplant diabetes mellitus in pediatric renal transplant recipients: a report of the North American Pediatric Renal Transplant Cooperative Study (NAPRTCS). Transplantation.

[19] Porrini EL, Díaz JM, Moreso F, Delgado Mallén PI, Silva Torres I, Ibernon M (2016). Clinical evolution of post-transplant diabetes mellitus. Nephrol Dial Transplant.

[20] Pham PT, Pham PM, Pham SV, Pham PA, Pham PC (2011). New onset diabetes after transplantation (NODAT) an overview. Diabetes Metab Syndr Obes.

[21] Hjelmesaeth J, Asberg A, Müller F, Hartmann A, Jenssen T (2005). New-onset posttransplantation diabetes mellitus: insulin resistance or insulinopenia? Impact of immunosuppressive drugs, cytomegalovirus and hepatitis C virus infection. Curr Diabetes Rev.

[22] Armstrong KA, Prins JB, Beller EM, Campbell SB, Hawley CM, Johnson DW, Isbel NM (2006). Should an oral glucose tolerance test be performed routinely in all renal transplant recipients?. Clin J Am Soc Nephrol.

[23] Yates CJ, Fourlanos S, Colman PG, Cohney S (2013). Screening for new-onset diabetes after kidney transplantation: limitations of fasting glucose and advantages of afternoon glucose and glycated hemoglobin. Transplantation.

[24] Zielińska K, Kukulski L, Wróbel M, Przybyłowski P, Zielińska M, Strojek K (2020). New Onset Diabetes After Transplantation (NODAT) — scientific data review. Clin Diabetol.

[25] Vincenti F, Friman S, Scheuermann E, Rostaing L, DIRECT (Diabetes Incidence after Renal Transplantation: Neoral C Monitoring Versus Tacrolimus) Investigators (2007). Results of an international, randomized trial comparing glucose metabolism disorders and outcome with cyclosporine versus tacrolimus. Am J Transplant.

[26] Dienemann T, Fujii N, Li Y, Govani S, Kosaraju N, Bloom RD, Feldman HI (2016). Long-term patient survival and kidney allograft survival in post-transplant diabetes mellitus: a single-center retrospective study. Transpl Int.

[27] Romagnoli J, Citterio F, Violi P, Cadeddu F, Nanni G, Castagneto M (2005). Post-transplant diabetes mellitus: a case-control analysis of the risk factors. Transpl Int.

[28] Burroughs TE, Lentine LK, Takemoto SK, Swindle J, Machnicki G, Hardinger K, Brennan DC, Irish WD, Schnitzler MA (2007). Influence of early post-transplantation prednisone and calcineurin inhibitor dosages on the incidence of new-onset diabetes. Clin J Am Soc Nephrol.

