# Supplementary material for: Monitoring of blood glucose after pediatric kidney transplantation: a longitudinal cohort study
Source: Pediatr Nephrol. 2022 Jul 11;38(3):847–58. doi: 10.1007/s00467-022-05669-0 (PMC9842551; doi:10.1007/s00467-022-05669-0)
Supplement: Supplementary file 1 — (PPTX 111 kb) [file 467_2022_5669_MOESM1_ESM.pptx]

## Slide 1
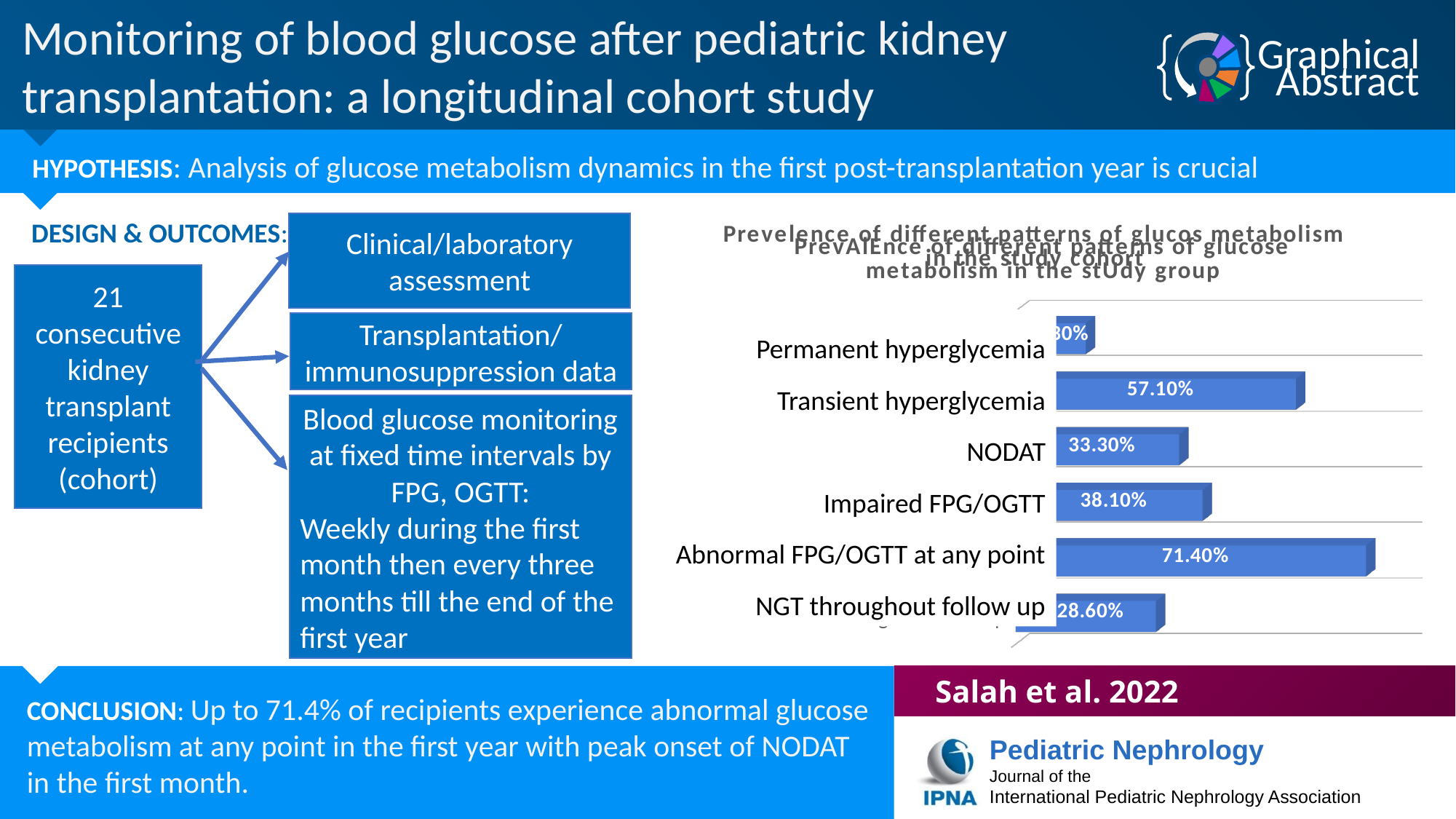

Monitoring of blood glucose after pediatric kidney
 transplantation: a longitudinal cohort study
HYPOTHESIS: Analysis of glucose metabolism dynamics in the first post-transplantation year is crucial
[unsupported chart]
DESIGN & OUTCOMES:
Clinical/laboratory assessment
[unsupported chart]
21 consecutive kidney transplant recipients (cohort)
Permanent hyperglycemia
Transient hyperglycemia
NODAT
Impaired FPG/OGTT
Abnormal FPG/OGTT at any point
NGT throughout follow up
Transplantation/ immunosuppression data
Blood glucose monitoring at fixed time intervals by FPG, OGTT:
Weekly during the first month then every three months till the end of the first year
 Salah et al. 2022
CONCLUSION: Up to 71.4% of recipients experience abnormal glucose metabolism at any point in the first year with peak onset of NODAT in the first month.
