# Supplementary material for: Monitoring of blood glucose after pediatric kidney transplantation: a longitudinal cohort study
Source: Pediatr Nephrol. 2022 Jul 11;38(3):847–58. doi: 10.1007/s00467-022-05669-0 (PMC9842551; doi:10.1007/s00467-022-05669-0)
Supplement: Supplementary file 2 — Supplementary file2 (DOCX 17 KB) [file 467_2022_5669_MOESM2_ESM.docx]

**Table (4):** Individual glycemic status at different points throughout the follow up

|  | **1 week** | **2 week** | **3 week** | **1 mo** | **3 mo** | **6 mo** | **9 mo** | **1 year** |
| --- | --- | --- | --- | --- | --- | --- | --- | --- |
| **Patient 1** | NGT | **DM** | NGT | NGT | NGT | NGT | NGT | NGT |
| **Patient 2** | **IFG,IGT** | NGT | NGT | NGT | NGT | **IFG,IGT** | **IFG,IGT** | **IFG,IGT** |
| **Patient 3** | NGT | NGT | NGT | **IFG** | **IFG** | NGT | NGT | NGT |
| **Patient 4** | NGT | NGT | NGT | NGT | **IFG,IGT** | **IFG,IGT** | **IFG,IGT** | **IFG** |
| **Patient 5** | NGT | NGT | NGT | NGT | NGT | NGT | NGT | NGT |
| **Patient 6** | NGT | NGT | NGT | NGT | NGT | NGT | NGT | NGT |
| **Patient 7** | NGT | NGT | NGT | NGT | **IGT** | NGT | NGT | NGT |
| **Patient 8*** | **DM** | **DM** | **IFG** | NGT | NGT | **IFG** | **IFG** | NGT |
| **Patient 9** | NGT | NGT | NGT | NGT | NGT | NGT | **IGT** | **IGT** |
| **Patient 10** | NGT | **IGT** | NGT | NGT | **IGT** | **IGT** | NGT | NGT |
| **Patient 11** | NGT | NGT | NGT | NGT | NGT | NGT | NGT | NGT |
| **Patient 12** | NGT | NGT | NGT | NGT | NGT | NGT | NGT | NGT |
| **Patient 13** | NGT | NGT | NGT | NGT | **IFG,IGT** | NGT | NGT | NGT |
| **Patient 14*** | NGT | **DM** | **DM** | **DM** | **IFG,IGT** | **IGT** | NGT | NGT |
| **Patient 15** | NGT | NGT | NGT | NGT | NGT | NGT | NGT | NGT |
| **Patient 16** | NGT | **DM** | **DM** | **IGT** | NGT | NGT | NGT | NGT |
| **Patient 17** | **IFG,IGT** | NGT | NGT | **IFG** | NGT | **IGT** | NGT | NGT |
| **Patient 18*** | **DM** | **DM** | **IFG,IGT** | **IFG** | NGT | NGT | NGT | NGT |
| **Patient 19** | NGT | NGT | NGT | NGT | NGT | NGT | NGT | NGT |
| **Patient 20** | NGT | **DM** | **IGT** | **IGT** | NGT | NGT | NGT | NGT |
| **Patient 21** | NGT | **DM** | NGT | NGT | NGT | NGT | NGT | NGT |

NGT (normal glucose tolerance), IFG (impaired fasting glucose), IGT (impaired glucose tolerance), Controlled on insulin * NODAT on insulin therapy
